# Temporal Trends and Patterns in Mortality After Incident Heart Failure: A Longitudinal Analysis of 86 000 Individuals

**DOI:** 10.1001/jamacardio.2019.3593

**Published:** 2019-09-03

**Authors:** Nathalie Conrad, Andrew Judge, Dexter Canoy, Jenny Tran, Ana-Catarina Pinho-Gomes, Elizabeth R. C. Millett, Gholamreza Salimi-Khorshidi, John G. Cleland, John J. V. McMurray, Kazem Rahimi

**Affiliations:** 1 The George Institute for Global Health, University of Oxford, Oxford, England; 2 Nuffield Department of Orthopaedics, Rheumatology and Musculoskeletal Sciences, University of Oxford, Nuffield Orthopaedic Centre, Oxford, England; 3 Bristol National Institute for Health Research Biomedical Research Centre, Musculoskeletal Research Unit, University of Bristol, Southmead Hospital, Bristol, England; 4 Medical Research Council Lifecourse Epidemiology Unit, University of Southampton, Southampton General Hospital, Southampton, England; 5 National Institute for Health Research Oxford Biomedical Research Centre, University of Oxford, Oxford, England; 6 Deep Medicine, Oxford Martin School, University of Oxford, Oxford, England; 7 Faculty of Medicine, University of New South Wales, Sydney, Australia; 8 Robertson Centre for Biostatistics and Clinical Trials, University of Glasgow and National Heart & Lung Institute, Imperial College London, London, England; 9 Institute of Cardiovascular and Medical Sciences, University of Glasgow, Glasgow, Scotland; 10 Oxford University Hospitals NHS Foundation Trust, Oxford, England

## Abstract

**Importance:**

Despite considerable improvements in heart failure care, mortality rates among patients in high-income countries have changed little since the early 2000s. Understanding the reasons underlying these trends may provide valuable clues for developing more targeted therapies and public health strategies.

**Objective:**

To investigate mortality rates following a new diagnosis of heart failure and examine changes over time and by cause of death and important patient features.

**Design, Setting, and Participants:**

This population-based retrospective cohort study analyzed anonymized electronic health records of individuals who received a new diagnosis of heart failure between January 2002 and December 2013 who were followed up until December 2014 from the Clinical Practice Research Datalink, which links information from primary care, secondary care, and the national death registry from a subset of the UK population. The data were analyzed from January 2018 to February 2019.

**Main Outcomes and Measures:**

All-cause and cause-specific mortality rates at 1 year following diagnosis. Poisson regression models were used to calculate rate ratios (RRs) and 95% confidence intervals comparing 2013 with 2002, adjusting for age, sex, region, socioeconomic status, and 17 major comorbidities.

**Results:**

Of 86 833 participants, 42 581 (49%) were women, 51 215 (88%) were white, and the mean (SD) age was 76.6 (12.6) years. While all-cause mortality rates declined only modestly over time (RR comparing 2013 with 2002, 0.94; 95% CI, 0.88-1.00), underlying patterns presented explicit trends. A decline in cardiovascular mortality (RR, 0.73; 95% CI, 0.67-0.80) was offset by an increase in noncardiovascular deaths (RR, 1.22; 95% CI, 1.11-1.33). Subgroup analyses further showed that overall mortality rates declined among patients younger than 80 years (RR, 0.79; 95% CI, 0.71-0.88) but not among those older than 80 years (RR, 0.97; 95% CI, 0.90-1.06). After cardiovascular causes (898 [43%]), the major causes of death in 2013 were neoplasms (311 [15%]), respiratory conditions (243 [12%]), and infections (13%), the latter 2 explaining most of the observed increase in noncardiovascular mortality.

**Conclusions and Relevance:**

Among patients with a new heart failure diagnosis, considerable progress has been achieved in reducing mortality in young and middle-aged patients and cardiovascular mortality across all age groups. Improvements to overall mortality are hindered by high and increasing rates of noncardiovascular events. These findings challenge current research priorities and management strategies and call for a greater emphasis on associated comorbidities. Specifically, infection prevention presents as a major opportunity to improve prognosis.

## Key Points

**Question:**

Why has there been no improvement in the prognosis for patients with heart failure over the past 15 years when considerable advances in heart failure care have been introduced during the same period?

**Findings:**

In this cohort study of patients who received a new diagnosis of heart failure between 2002 and 2013 in the United Kingdom, cardiovascular mortality declined by 27% and premature deaths from any cause declined by 21%. Improvements to overall mortality were hindered by noncardiovascular diseases, which represented most deaths and increased by 22% over time.

**Meaning:**

Management strategies that solely target cardiovascular outcomes appear insufficient to improve the survival of patients with heart failure; the management of associated comorbidities, particularly infection prevention, appears as a major priority and opportunity.

## Teaser

This cohort study examines trends in outcomes and mortality rates for UK patients with a new heart failure diagnosis.

## Introduction

The past 25 years have brought considerable improvements in heart failure care. New treatments, such as β-blockers,^1^ mineralocorticoid receptor antagonists,^2^ ivabradine,^3^ and sacubitril/valsartan,^4^ and device therapies, such as implantable cardioverter defibrillators^5^ and cardiac resynchronization therapy,^6^ have been introduced. Multidisciplinary management teams, including specialist nurses, have also been developed to improve the delivery of care.^7,8^ Randomized clinical trials have demonstrated the effectiveness of these treatments in reducing mortality and hospitalizations, and observational studies have shown that they are increasingly being used worldwide.^9,10,11^ Despite this, several recent studies have reported that the decline in mortality rates among patients with heart failure has been stalling since the mid-2000s.^12,13,14^ To our knowledge, the reasons underlying this apparent paradox are unknown.

Most studies that have investigated outcomes following incident heart failure have restricted analyses to all-cause mortality without investigating underlying patterns, such as changes by subgroup or cause-specific mortality (eTable 1 in the Supplement). In addition, information about trends in cause-specific hospitalization rates, which are highly important to patients and clinicians, remain poorly investigated. In-depth analyses investigating how specific causes of morbidity and mortality and changes in patient characteristics contribute to overall trends would complement these efforts and may provide valuable clues for the development of more targeted therapies or public health strategies.

To address these knowledge gaps, we used a database of electronic health records that links information from primary care, secondary care, and the national death registry for a representative sample of the UK population. We performed a detailed assessment of health outcomes in patients with a new diagnosis of heart failure and analyzed changes in cause-specific mortality and morbidity over time and by important patient characteristics, such as age, sex, and socioeconomic status.

## Methods

### Data Source

We used electronic health records from the Clinical Practice Research Datalink (CPRD) from January 1, 1985, to September 30, 2015. The CPRD database contains anonymized patient data from approximately 7% of the current UK population and is broadly representative in terms of age, sex, and race/ethnicity.^15^ The CPRD is one of the largest databases of longitudinal medical records in the world and has been validated for epidemiological research for a range of conditions.^15^ Primary care records were linked to hospital admissions using Hospital Episodes Statistics Admitted Patient Care data and mortality data from the Office for National Statistics. Scientific approval for this study was given by the CPRD independent scientific advisory committee and, as an observational study using anonymized data, was exempt from the requirement for patient consent.

### Study Population

The study was restricted to records of acceptable quality^15^ and approved for Hospital Episodes Statistics and Office for National Statistics linkage. Patients eligible for inclusion in the study were men and women 16 years and older who were registered with their general practice for at least 12 months. We defined *incident heart failure* as the first record of heart failure in primary care or hospital admission records from any diagnostic position using a comprehensive set of diagnostic codes (eTable 2 in the Supplement) and following previously published methods.^16^ For those who received their diagnosis in the hospital, the date of diagnosis was set to the date of discharge. We identified all incident heart failure cases from January 1, 2002, to December 31, 2013, and excluded individuals whose first diagnosis referred to a preexisting condition (eTable 3 in the Supplement) or was recorded before the study start date (January 1, 2002) or within the first 12 months of registration with the general practice.

### Study Outcomes

We investigated mortality rates at 1 year following incident diagnosis as well as the number of hospital admissions with an overnight stay within 1 year of incident diagnosis (not counting index admission for those who received their diagnosis in the hospital). The *cause of death* was defined as the first reported cause in each patient’s death certificate. The *cause of hospitalizatio*n was defined as the primary discharge diagnosis. Causes of death and hospitalization were mapped to 9 and 11 disease categories, respectively (eAppendix 1 in the Supplement). In subgroup analyses, disease categories were further grouped into cardiovascular and noncardiovascular causes (eTable 4 in the Supplement).

### Baseline Variables

We extracted baseline characteristics from patients’ health records, including socioeconomic status, systolic and diastolic blood pressure, smoking status, body mass index (calculated as weight in kilograms divided by height in meters squared), and the prevalence of 17 comorbidities (eAppendix 2 in the Supplement). Baseline characteristics are presented as frequencies (percentage) for categorical data, medians and interquartile ranges for non-normally distributed continuous data, or mean (SD) for normally distributed continuous data.

### Statistical Analysis

We report crude mortality and hospitalization rates as well as adjusted rate ratios (RRs) by calendar year of diagnosis and subgroups (age, sex, socioeconomic status, and place of diagnosis). Crude mortality rates were computed as the cumulative incidence of mortality at 1 year accounting for observation time. Cause-specific mortality rates were computed accounting for the competing risk of death from other causes.^17^ Hospitalizations were assessed as the number of hospital admissions per patient-years of follow-up within 1 year of heart failure diagnosis.

To examine trends over time and by subgroup, we used Poisson regression models offset for observation time and present resulting rate ratios and corresponding 95% confidence intervals. All models account for the calendar year of diagnosis, age at diagnosis (as a continuous variable), sex, region, socioeconomic status, and baseline comorbidities. Follow-up time was considered from the date of incident heart failure diagnosis up to the earliest of the following dates: patient died, patient deregistered from their practice, or the practice ceased contributing data, and for a maximum of 1 year.

To assess the robustness of observed temporal trends, we grouped the first 3 and the last 3 years of the study together and assessed whether the direction and statistical significance of trends were similar to those reported in the main analyses. The study findings are reported in accordance with the Reporting of Studies Conducted Using Observational Routinely Collected Health Data recommendations.^18^ Statistical analyses were performed in R, version 3.4.2 (R Foundation) and statistical significance was set at *P *< .05.

## Results

We identified 86 833 patients who developed incident heart failure from 2002 to 2013. At the time of diagnosis, 41 88 patients (48%) were 80 years or older, 42 581 (49.0%) were women, and 68 451 (79%) had 3 or more comorbidities. Over the study period, we observed a modest increase in patients’ age at diagnosis, a marked increase in multimorbidity, and a greater proportion of patients who received diagnoses in secondary care settings as opposed to primary care (Table).

**Table.** Baseline Characteristics of Patients With Incident Heart Failure in CPRD From 2002 to 2013^a^

Characteristic	No. (%)		
	Full Cohort (N = 86 833)	Period	
		2002-2004 (n = 21 943 [25%])	2011-2013 (n = 22 065 [25%])
Age, mean (SD), y	76.6 (12.6)	76.5 (12.1)	76.9 (12.9)
Age ≥ 80 y	41 888 (48)	10 129 (46)	11 079 (50)
Women	42 581 (49)	10 889 (50)	10 718 (49)
Race/ethnicity			
White	51 215 (88)	12 038 (92)	16 219 (87)
Missing	28 316 (33)	8912 (41)	3431 (16)
Socioeconomic status			
1 (Least deprived)	17 024 (20)	4177 (19)	4514 (20)
2	18 680 (22)	4680 (21)	4808 (22)
3	18 709 (22)	4769 (22)	4734 (21)
4	17 149 (20)	4387 (20)	4230 (19)
5 (Most deprived)	15 271 (18)	3930 (18)	3779 (17)
Systolic blood pressure			
Mean (SD), mm Hg	133 (21)	137 (24)	130 (19)
Missing	5080 (6)	2601 (12)	682 (3)
Diastolic blood pressure			
Mean (SD), mm Hg	75 (11)	77 (12)	73 (11)
Missing	5192 (6)	2601 (12)	705 (3)
BMI^b^ category			
Underweight	2022 (4)	329 (3)	611 (4)
Normal	16 090 (31)	3000 (31)	4632 (31)
Overweight	17 397 (34)	3434 (35)	4889 (32)
Obesity	9742 (19)	1824 (19)	2872 (19)
Severe obesity	6440 (12)	1086 (11)	2080 (14)
Missing	35 142 (40)	12 270 (56)	6981 (32)
Smoking			
No	27 405 (41)	5081 (41)	7377 (41)
Former	30 191 (45)	5192 (42)	8416 (46)
Yes	9002 (14)	2031 (17)	2320 (13)
Missing	20 235 (23)	9639 (44)	3952 (18)
Diagnosis setting			
Primary care	38 448 (44)	11 952 (54)	8266 (37)
Hospital admission, HF primary cause	10 838 (12)	2650 (12)	2588 (12)
Hospital admission, HF secondary cause	37 547 (43)	7341 (33)	11 211 (51)
Cardiovascular comorbidities			
Atrial fibrillation	34 048 (39)	6990 (32)	9884 (45)
Hypertension	57 639 (66)	11 938 (54)	16 540 (75)
Ischemic heart disease	42 513 (49)	10 279 (47)	11 032 (50)
Peripheral artery disease	12 562 (14)	2754 (13)	3432 (16)
Stroke	16 186 (19)	3893 (18)	4389 (20)
Respiratory comorbidities			
Asthma	20 119 (23)	4366 (20)	5867 (27)
Chronic obstructive pulmonary disease	16 560 (19)	3782 (17)	4720 (21)
Other comorbidities			
Anemia	22 277 (26)	4187 (19)	6987 (32)
Cancer	21 512 (25)	4360 (20)	6466 (29)
Chronic kidney disease	20 526 (24)	1370 (6)	7940 (36)
Dementia	5203 (6)	1003 (5)	1689 (8)
Depression	18 663 (21)	3963 (18)	5507 (25)
Diabetes	18 847 (22)	3893 (18)	5465 (25)
Dyslipidemia	23 486 (27)	3360 (15)	8253 (37)
Obesity	10 729 (12)	1659 (8)	3772 (17)
Osteoarthritis	37 039 (43)	7960 (36)	10 702 (49)
Thyroid disorder	10 401 (12)	2099 (10)	3027 (14)
Three or more comorbidities	68 451 (79)	14 876 (68)	19 018 (86)

Abbreviations: BMI, body mass index; CPRD, Clinical Practice Research Datalink; HF, heart failure; IMD, Index Multiple Deprivation.

^a^ Number and percentage of records with missing data are displayed for variables with missing entries. Category percentages refer to complete cases. Socioeconomic status refers to Index of Multiple Deprivation 2015 quintile, with 1 referring to the most affluent and 5 to the most deprived quintile. Number of comorbidities refers to any of the 17 conditions investigated.

^b^ Calculated as weight in kilograms divided by height in meters squared.

### Mortality

**Figure 1.**
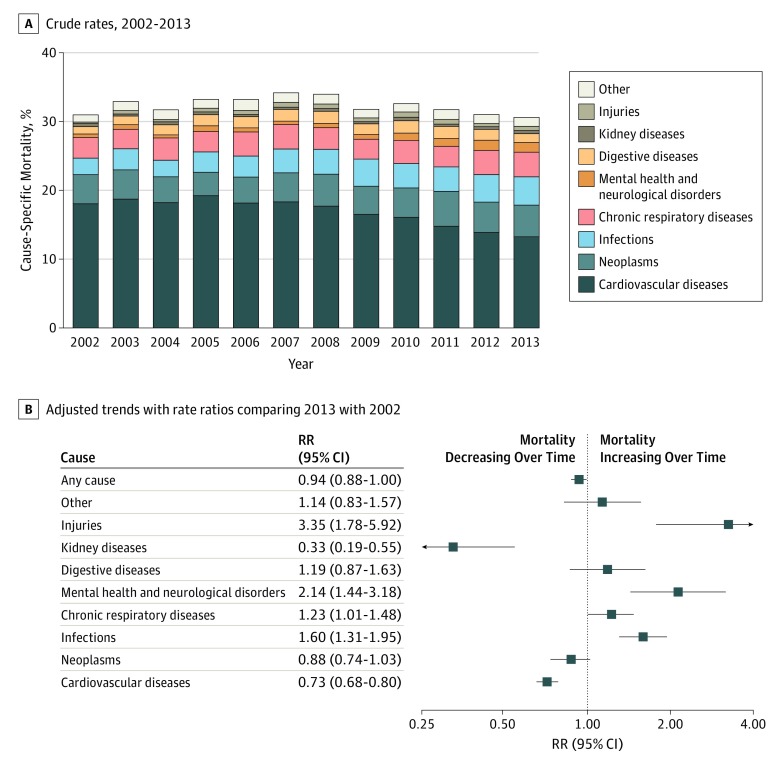
Temporal Trends in All-Cause and Cause-Specific Mortality Rates at 1 Year Following Incident Heart Failure A, Crude rates of all-cause and cause-specific mortality at 1 year following incident heart failure diagnosis. Labels for years 2002 and 2013 present individual causes of death as a share of the total number of deaths at 1 year. B, Rate ratios (RRs) from multivariable Poisson regression models comparing 1-year mortality rates in 2013 with 2002 by first reported cause, adjusting for patients’ age, sex, socioeconomic status, region, and 17 baseline comorbidities.

One-year mortality rates following incident heart failure were high (32%) and declined only modestly over the period of study (adjusted RR comparing 2013 with 2002, 0.94; 95% CI, 0.88-1.00). When overall mortality rates were stratified by specific causes, diverging trends between death from cardiovascular and noncardiovascular causes became apparent. One-year mortality rates from cardiovascular causes declined from 18% in 2002 to 13% in 2013 (RR 2013 vs 2002, 0.73; 95% CI, 0.67-0.80), whereas noncardiovascular mortality rates increased over the same period from 13% to 17% (RR 2013 vs 2002, 1.22; 95% CI, 1.11-1.33). Among patients who died in 2013, the most frequent causes of death after cardiovascular diseases (43% of all deaths) were neoplasms (15%), infections (13%), and chronic respiratory conditions (12%). Deaths associated with infections, chronic respiratory diseases, injuries, and mental health or neurological disorders increased during the period of study (Figure 1). Mortality due to infections accounted for the largest absolute increase over time, representing 173 (8%) and 279 (13%) deaths in 2002 and 2013, respectively (RR 2013 vs 2002, 1.60; 95% CI, 1.31-1.95). Further analyses of individual causes of death revealed influenza and pneumonia as important and increasing causes of death (1915 deaths [6%] at 1 year; RR 2013 vs 2002, 1.59; 95% CI, 1.26-1.99), now accounting for about as many deaths as myocardial infarction and more deaths than cerebrovascular disease. Although mortality from chronic respiratory diseases was largely due to chronic obstructive pulmonary disease, the increase over time in this category was attributable to interstitial lung diseases, which represented 13 (1%) and 37 (2%) deaths in 2002 and 2013, respectively (RR 2013 vs 2002, 3.37; 95% CI, 1.77-6.42). Finally, deaths from injuries were most commonly associated with falls or unspecified incidental causes and deaths attributed to mental health and neurological disorders were largely due to dementia, including Alzheimer disease (eTable 5 in the Supplement).

**Figure 2.**
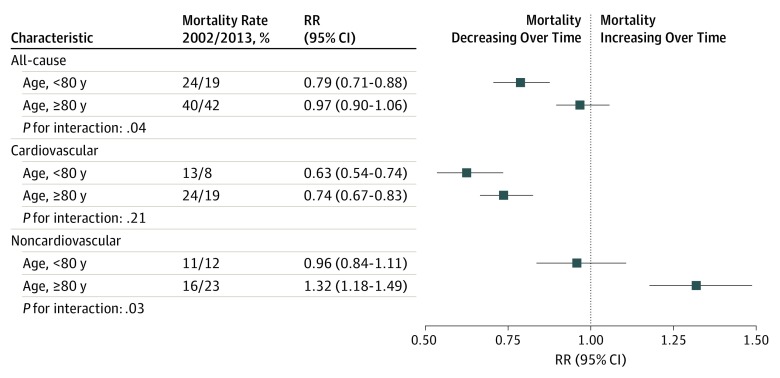
Temporal Trends in All-Cause and Cause-Specific Mortality Rates at 1 Year Following Incident Heart Failure by Age Group Crude mortality rates by age-groups alongside rate ratios (RRs), 95% confidence intervals, and interaction *P* values from multivariable Poisson regression models accounting for year of diagnosis, age (as a continuous variable), sex, socioeconomic status, region, and 17 baseline comorbidities. Interaction *P* values refer to the interaction between age group (categorized as age <80 years or age ≥80 years) and year of diagnosis.

Age-stratified analyses further revealed diverging trends over time: all-cause mortality declined among patients younger than 80 years (RR 2013 vs 2002, 0.79; 95% CI, 0.71-0.88) but not in older individuals (RR 2013 vs 2002, 0.97; 95% CI, 0.9-1.06). Cause-specific analyses showed that cardiovascular mortality declined across all age groups, although less steeply in older age groups, and that the increase in noncardiovascular mortality was largely attributable to older age groups (Figure 2). Individual causes of death also differed by age. Specifically, digestive diseases (in particular liver cirrhosis) and neoplasms were more common in younger patients, whereas infections and mental health or neurological disorders were more common in older age groups (eFigure 1 in the Supplement).

**Figure 3.**
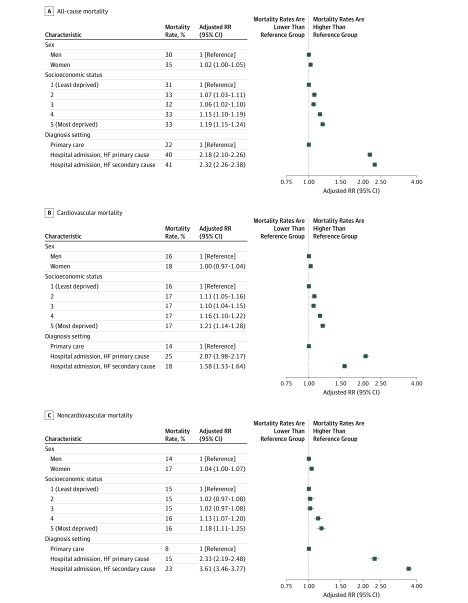
Differences in Mortality Rates 1 Year After Incident Heart Failure (HF) by Sex, Socioeconomic Status, and Diagnosis Care Setting Crude mortality rates by subgroups alongside rate ratios (RRs) and 95% confidence intervals from multivariable Poisson regression models accounting for year of diagnosis, age (as a continuous variable), sex, socioeconomic status, region, and 17 baseline comorbidities. Socioeconomic status refers to Index of Multiple Deprivation 2015 quintile, with 1 referring to the most affluent and 5 to the most deprived socioeconomic quintile.

While overall mortality was similar in men and women (Figure 3), causes of death presented sex-specific patterns. Differences were particularly apparent among patients younger than 65 years regarding cardiovascular causes (more prominent in men), cancer, and infections (more prominent in women) (eFigure 1 in the Supplement).

Finally, patients’ socioeconomic background and the care setting in which patients first received their diagnoses were important predictors of health outcomes. For the same age and sex, patients from more deprived socioeconomic backgrounds had 19% higher mortality rates than their more affluent counterparts (RR for most deprived vs least deprived quintile, 1.19; 95% CI, 1.15-1.24; Figure 3). One-year mortality was also higher in patients who received their heart failure diagnosis in the hospital (19 167 [41%]) compared with those receiving their diagnosis in primary care (8231 [22%]) (RR for hospital vs primary care diagnoses, 2.29; 95% CI, 2.23-2.35; Figure 3). Among those who received their diagnosis in the hospital, 10 302 (21%) died before discharge, and rates did not change significantly over the study period (RR 2013 vs 2002, 0.91; 95% CI, 0.82-1.01).

### Hospitalizations

**Figure 4.**
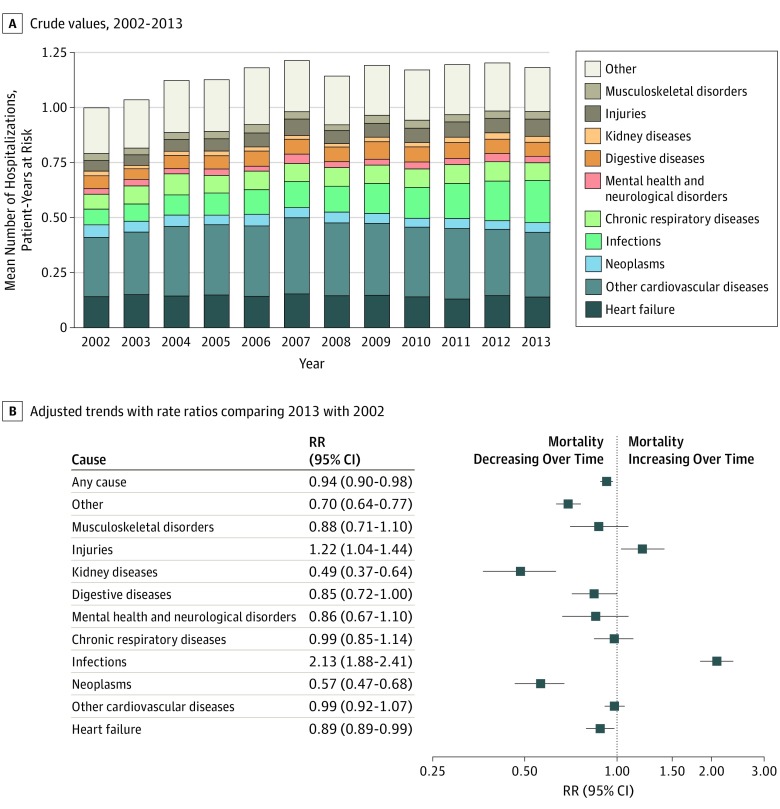
Temporal Trends in All-Cause and Cause-Specific Mean Number of Hospitalizations at 1 Year Following Incident Heart Failure A, Crude mean number of hospitalizations within 1 year of incident heart failure per patient-years at risk by primary discharge diagnosis. Labels displayed for 2002 and 2013 present individual discharge diagnoses as a share of the total number of hospitalizations in the given year. B, Rate ratios (RRs) from multivariable Poisson regression models comparing mean number of hospitalizations at 1 year in 2013 with 2002 offset for patient-years at risk by primary discharge diagnosis, adjusting for age, sex, socioeconomic status, region, and 17 baseline comorbidities.

The number of hospital admissions in the year following incident heart failure was high (1.15 hospitalizations per patient-year at risk). Although crude rates increased by 20% over time, adjusted rates accounting for patient characteristics and comorbidities at baseline declined by 6% over the study period (RR 2013 vs 2002, 0.94; 95% CI, 0.90-0.98; Figure 4).

Admissions for heart failure represented 13% of hospitalizations within a year of diagnosis (0.14 hospitalizations per patient-year at risk) and showed a relative decline of 11% over the study period (RR 2013 vs 2002, 0.89; 95% CI, 0.80-0.99). Overall, hospitalizations associated with any cardiovascular reason (eg, heart failure or another cardiovascular disorder) represented fewer than half of all admissions and did not change significantly over time (0.46 hospitalizations per patient-year at risk; RR 2013 vs 2002, 0.96; 95% CI, 0.91-1.03). In parallel, admissions for some noncardiovascular causes, in particular infections and injuries, increased (Figure 4).

Age-stratified patterns of hospital admissions were similar to those reported for mortality. The number of admissions declined among patients younger than 80 years (RR 2013 vs 2002, 0.82; 95% CI, 0.77-0.88) but not in older individuals (RR 2013 vs 2002, 1.12; 95% CI, 1.04-1.21). Differences by sex, socioeconomic status, and place of diagnosis were also consistent, although less pronounced than those reported for mortality (eFigures 2 and 3 in the Supplement).

## Discussion

This study reveals possible explanations for the standstill in mortality observed among patients with heart failure in many high-income countries. Important improvements in cardiovascular mortality have been offset by a large and increasing number of deaths due to noncardiovascular disorders, such as infections and respiratory problems. Overall survival rates improved in younger and middle-aged patients as a result of fewer cardiovascular deaths, yet mortality changed little in patients 80 years or older who comprised almost half of all heart failure cases. Broadly similar findings were seen for hospital admissions.

The temporal trends in all-cause mortality rates observed in this study align with earlier reports from high-income countries that have also shown little change since the mid-2000s (eTable 1 in the Supplement). Previous studies have rarely investigated underlying causes of death or hospitalizations and had limited ability to adjust for other concomitant changes, such as the rise in comorbidities over time. To our knowledge, the only study that has reported cardiovascular and noncardiovascular mortality and morbidity trends separately was the Olmsted county study, which was limited by sample size and statistical power to make robust conclusions about changing patterns.^12^ Complementing these earlier studies, we found that within the last 12 years, the relative risk of death from cardiovascular causes after incident diagnosis of heart failure has declined by 27%. However, this important improvement in outcomes was offset by a 22% increase in noncardiovascular death rates. While the increasing burden of multimorbidity in patients with heart failure may have contributed to the observed changes in causes of death, our analyses show that the increase in noncardiovascular events remains significant after adjusting for 17 major comorbidities, and hence suggest that other factors contribute.

Current heart failure treatment is intrinsically disease-centered and essentially focused on cardiac dysfunction and its consequences. Cardiovascular mortality and admissions for heart failure are key indicators of the effectiveness of heart failure–specific treatments. Thus, the decline in cardiovascular events is encouraging and appears to follow the introduction of national reporting and incentives schemes to improve evidence-based heart failure management^19,20^ and increased use of life-saving therapies, which have been observed during the study period.^9^ However, this study also revealed that noncardiovascular outcomes now account for most deaths and hospitalizations, a finding that is consistent with studies of unselected patient populations^12,14^ but represents a much higher share compared with reports from clinical trials.^21^ These findings challenge current research priorities and management strategies and have implications for the development of life-saving therapies.

Noncardiovascular comorbidities, hospitalizations, and deaths are in themselves an important potential therapeutic target in patients with heart failure. For example, infections appeared to represent the largest driver behind the recent increase in noncardiovascular mortality and hospitalizations we observed in this study. Most infection-associated deaths were due to influenza and pneumonia and some of those may have been preventable through better care. For instance, the coverage of influenza vaccination among patients with heart failure in the United Kingdom, although high compared with many other countries,^22^ has been declining,^23^ which could have contributed to the observed trend.

Chronic respiratory conditions, injuries, and dementia further contributed to the increasing rates of noncardiovascular mortality, yet with a more modest association with overall burden. The observed increase in mortality from chronic respiratory conditions was largely attributable to interstitial lung diseases. This finding is consistent with reports of increasing rates of detection, incidence, and mortality associated with interstitial lung diseases in the general population, in the United Kingdom, and worldwide^24,25,26^ and may, therefore, not be specific to heart failure. Nonetheless, patients with heart failure often receive treatments known to cause pulmonary fibrosis, including antibiotics, amiodarone, and repeated exposure to therapeutic oxygen.^27,28^ Further studies are needed to fully understand the reasons for the increasing rates of interstitial lung diseases among patients with heart failure and guide clinical decision-making in patients at high risk of pulmonary complications.

Falls are common in elderly populations^29^ and the perception that the blood pressure–lowering effects of heart failure therapies may place patients at even higher risk sometimes creates a barrier to effectively treating patients with heart failure in the community.^30,31^ This study quantifies the long-term association of injuries with patients with heart failure over a period of time that has witnessed a gradual increase in the use of blood pressure–lowering therapies^9^ and shows that while rates of injuries in this cohort have increased over time, their contribution to overall mortality and morbidity in patients with heart failure remains relatively modest (2% of deaths and 7% of hospitalizations are associated with injuries). Hence these findings do not support the perception that falls present a major health burden among patients with heart failure. Nevertheless, strategies to avoid falls and prevent injuries are an appropriate focus in these patients, in particular in the context of high rates of osteoporosis in this patient group.^32^

Our age-stratified analyses provide additional insights into underlying mechanisms and opportunities to improve patient care. Declining rates of cardiovascular mortality across all age groups show that progress in cardiovascular care has benefitted young and old. However, unchanged survival among older patients highlights the importance of multimorbidity, frailty, and senescence rather than cardiac dysfunction as important determinant of prognosis. With about 80% of patients with heart failure having multiple comorbidities and almost 50% being 80 years or older at the time of diagnosis, it appears crucial to better understand the needs of patients encountered in usual care and to reassess research objectives and therapeutic options accordingly.

### Strengths and Limitations

A strength of this study is the large patient cohort studied, providing sufficient cases for cause-specific analyses, and the long period of study, which allowed study of long-term trends. This population-based cohort also reflects patients as encountered in routine care so that findings are likely to be more broadly generalizable compared with surveys or clinical trials that enroll selected participants. One of the key limitations of this study was the relatively limited clinical information contained in electronic health records. In particular, left ventricular ejection fraction measurements were not available and we were unable to stratify analyses by type of heart failure. While this limitation is important, particularly as heart failure treatments have only been demonstrated to be effective in patients with heart failure and reduced ejection fraction, evidence from several large-scale observational studies shows that mortality rates and trends over time do not differ significantly between patients with preserved or reduced ejection fraction.^12,33^ Moreover, the current evidence base does not suggest that the case-mix of patients with newly diagnosed heart failure would have shifted significantly toward one or the other type of ejection fraction over the study period.^12^ Another limitation of this study is that we are unable to make any direct inference on the actual association of adherence with guideline-recommended medical treatment toward the observed temporal changes in deaths and hospitalizations. Further limitations arise from to the limited information available to adjust for disease severity at baseline or the clinician (eg, specialty of admission ward). Finally, research using routinely collected health care data also rely on the accuracy of clinical coding input. The validity of clinical diagnoses recorded in UK primary care, secondary care, and death certificates CPRD has been independently investigated for a range of conditions and is generally considered appropriate for the purpose of this study (eAppendix 3-5 in the Supplement).

## Conclusions

Our findings have important implications for public health policies. Significant reductions in cardiovascular events and improved survival of patients with heart failure patients younger than 80 years attest to the progress made in patient care and encourage continued efforts to increase the use of evidence-based therapy. Further improvements in patient prognosis are likely to require a broader perspective on heart failure management, one that considers not only patients’ cardiovascular health but also the range of associated comorbidities and special needs of elderly patients.

## Notes

Supplement. eAppendix 1. Approach used to define disease categories for causes of deaths and hospitalizations eAppendix 2. Baseline variables eAppendix 3. Validity of diagnoses recorded in electronic health record databases eAppendix 4. Accuracy of hospital episodes data eAppendix 5. Validity of causes of death records eAppendix 6. Literature review eTable 1. Selected studies reporting heart failure mortality rates following incident heart failure eTable 2. Diagnostic codes that refer to a new diagnosis of heart failure eTable 3. Diagnostic codes that refer to pre-existing heart failure eTable 4. Definition of disease categories for causes of deaths and hospitalizations eTable 5. Number of deaths due to cardiovascular diseases, neoplasms, infections, and chronic respiratory diseases, by disease subgroup eTable 6. Clinical codes used to categorize causes of death or hospitalization as infections eTable 7. Clinical codes used to categorize causes of death or hospitalization as chronic respiratory diseases eFigure. All-cause and cause-specific mortality rates at 1-year following incident heart failure, by age and sex eReferences
