# Supplementary material for: Temporal Trends and Patterns in Mortality After Incident Heart Failure: A Longitudinal Analysis of 86 000 Individuals
Source: JAMA Cardiol. 2019 Sep 3;4(11):1102–11. doi: 10.1001/jamacardio.2019.3593 (PMC6724155; doi:10.1001/jamacardio.2019.3593)
Supplement: Supplement. — eAppendix 1. Approach used to define disease categories for causes of deaths and hospitalizations eAppendix 2. Baseline variables eAppendix 3. Validity of diagnoses recorded in electronic health record databases eAppendix 4. Accuracy of hospital episodes data eAppendix 5. Validity of causes of death records eAppendix 6. Literature review eTable 1. Selected studies reporting heart failure mortality rates following incident heart failure eTable 2. Diagnostic codes that refer to a new diagnosis of heart failure eTable 3. Diagnostic codes that refer to pre-existing heart failure eTable 4. Definition of disease categories for causes of deaths and hospitalizations eTable 5. Number of deaths due to cardiovascular diseases, neoplasms, infections, and chronic respiratory diseases, by disease subgroup eTable 6. Clinical codes used to categorize causes of death or hospitalization as infections eTable 7. Clinical codes used to categorize causes of death or hospitalization as chronic respiratory diseases eFigure. All-cause and cause-specific mortality rates at 1-year following incident heart failure, by age and sex eReferences [file jamacardiol-4-1102-s001.pdf]

Supplementary Online Content

Conrad N, Judge A, Canoy D. Temporal trends and patterns in mortality after incident heart failure: a longitudinal study of 86 000 individuals. *JAMA Cardiol*. Published online September 3, 2019. doi:10.1001/jamacardio.2019.3593

eAppendix 1. Approach used to define disease categories for causes of deaths and hospitalizations.

eAppendix 2. Baseline variables

eAppendix 3. Validity of diagnoses recorded in electronic health record databases

eAppendix 4. Accuracy of hospital episodes data

eAppendix 5. Validity of causes of death records

eAppendix 6. Literature review

eTable 1. Selected studies reporting heart failure mortality rates following incident heart failure

eTable 2. Diagnostic codes that refer to a new diagnosis of heart failure

eTable 3. Diagnostic codes that refer to pre-existing heart failure

eTable 4. Definition of disease categories for causes of deaths and hospitalizations

eTable 5. Number of deaths due to cardiovascular diseases, neoplasms, infections, and chronic respiratory diseases, by disease sugroup

eTable 6. Clinical codes used to categorize causes of death or hospitalization as infections

eTable 7. Clinical codes used to categorize causes of death or hospitalization as chronic respiratory diseases

e**Figure.** All-cause and cause-specific mortality rates at 1-year following incident heart failure, by age and sex

eFigure 2. Differences in hospital admissions within a year of incident heart failure, by sex, socioeconomic status and diagnosis care setting.

eFigure 3. Temporal trends in one-year mortality rates following incident heart failure, by age group.

eReferences

© 2019 Conrad N et al. *JAMA Cardiology*.

eAppendix 1: Approach used to define disease categories for causes of deaths and hospitalizations

The CPRD data used in this study provides causes of death and hospitalizations in the form of clinical codes from the ICD-10 (International Classification of Diseases 10th edition) system. We sought to categorize causes of death and hospitalizations into a set of unique and clinically meaningful disease categories. For that purpose, we used the following approach, which we applied to deaths and hospitalizations separately: (i) As a starting point, categories were defined as each code's overarching ICD chapter (n = 22); (ii) Chapters that presented similarities for the purpose of this study were grouped together (e.g. ICD chapters "Injury, poisoning and certain other consequences of external causes" and "External causes of morbidity and mortality" were grouped together and categorized as "Injuries"); (iii) Groups of conditions that, in 2013, represented at least 2% of deaths (for the categorization of causes of deaths) or either 2% of deaths or hospitalizations (for the categorization of causes of hospitalizations), e.g. kidney diseases or infections, were defined as independent categories; (iv) Any remaining category that represented less than 2% of deaths/hospitalizations in itself was classified as "other". In the UK, guidance for completing medical certificates states that heart failure is not a cause but a mode of death and discourages doctors from recording heart failure as the underlying cause of death.[1,2] Therefore, heart failure was defined as an individual disease category for hospitalizations, yet not for deaths.

eAppendix 2: Baseline variables

We extracted the most recent measurement of baseline characteristics recorded in patients primary care record within two years prior to incident heart failure diagnosis - these included systolic and diastolic blood pressure, smoking status, and body-mass index (BMI). BMI was categorized as underweight (<18·5 kg/m2), normal (18·5–24·9 kg/m2), overweight (25–29·9 kg/m2), obese (30–34·9 kg/m2), and severely obese (≥35 kg/m2).

We further report the care setting in which heart failure was first diagnosed. Diagnoses recorded during a hospital admission were further categorized based on whether heart failure was listed in primary or secondary diagnostic position. Diagnoses first recorded in primary care are likely to reflect both outpatient consultations by specialists and direct diagnoses by general practitioners.

To describe co-morbidities, we selected 17 common chronic conditions (anemia, asthma, atrial fibrillation, cancer, chronic kidney disease, chronic obstructive pulmonary disease, dementia, depression, diabetes, dyslipidemia, hypertension, ischemic heart disease, obesity, osteoarthritis, peripheral arterial disease, stroke, thyroid disease). Diagnosis code lists for the extraction of each condition were adapted from the CALIBER code repository.[3]

To describe socioeconomic status, we used the Index of Multiple Deprivation (IMD) 2015 quintile,[4] a composite measure of relative deprivation at a small area level, ranked in ascending order of deprivation score and grouped in equal fifths.

Ethnicity is reported as recorded in patient's electronic health record. When ethnicity differed between primary and secondary care records, secondary care data was used. To assist readability, ethnicity was grouped into two categories, 'white' and 'other'.

Baseline characteristics are presented as frequencies (%) for categorical data, medians and interquartile range (IQR) for non-normally distributed continuous data, or means and standard deviation (SD) for normally distributed continuous data.

eAppendix 3: Validity of diagnoses recorded in electronic health record databases

Research using electronic health records databases is reliant on the accuracy of clinical coding input by physicians in primary care, as part of a consultation, or secondary care, as part of a hospital admission. The validity of diagnoses underlying our study has therefore been carefully assessed and was considered appropriate in light of the following arguments.

Independent validation studies. Three studies are of major importance: (i) a systematic review, published in 2010, reports 212 validation studies over a broad range of conditions with an average positive predictive value of 89%[5]; (ii) a study specifically investigating heart failure diagnoses, which despite it being conducted before the introduction of national care monitoring programs reports a positive predictive value of 82%[6]; and (iii) a more recent study investigating the validity of chronic obstructive pulmonary disease (COPD), another major chronic condition managed in primary care, which reports an accuracy of 87% compared with specialist assessment.[7]

© 2019 American Medical Association. All rights reserved.

National care monitoring programs. Two national clinical audit programs (in particular the 'quality and outcomes framework' (QOF) introduced in 2004 for primary care, and the 'national heart failure audit' (NHFA) introduced in 2007 for secondary care) ensure a stable quality of clinical coding practices and provide a solid support for the validity of recorded diagnoses. Indeed, these report that approximately 90% of recorded heart failure diagnoses in England are referred for echocardiography, specialist assessment, or B-type natriuretic peptide (BNP) measurement.[8,9]

Clinical guidelines. Guidelines for the diagnosis and management of heart failure from the National Institute for Clinical Excellence (NICE)[10,11] provide additional consistency over the study period. Indeed, guidelines are largely consistent in regard to heart failure diagnostic criteria and recommended investigations. One important change is the availability of natriuretic peptides testing and the variability in assay accuracy; these are however mainly used to exclude suspected cases, as opposed to confirming diagnoses, and therefore unlikely to impact disease incidence rates.[10,11]

Sensitivity analyses. Finally, to confirm the validity of heart failure cases included in our cohort, we performed the following sensitivity analyses. (a) case identification restricted to diagnostic codes included in national care monitoring programs. While for our main analysis we intentionally expanded the diagnostic codes from the national audit programs list with additional codes indicating a heart failure diagnosis, so as to ensure completeness; sensitivity analyses, restricting diagnostic codes to those used in the national audit programs, found that 97% of patients in our cohort had a record heart failure used in the national clinical audit programs, and led to no significant changes in the present results. (b) case identification restricted to diagnoses recorded in secondary care, or referred for specialist assessment or echocardiography. We further found that 92% of patients included in our cohort had a heart failure diagnosis recorded in secondary care, or either a referral to specialist cardiology service or echocardiography. While that proportion moderately increased over time, we found no significant change by sex or socio-economic status.

eAppendix 4: Accuracy of hospital episodes data

Accuracy of diagnostic coding in routinely collected hospital records in the United Kingdom has been widely studied and findings show that data are sufficiently robust to be used in healthcare research and decision-making. Specifically, a recent systematic review identified 32 studies were that compared routinely collected data with case or operation notes. Although accuracy presented significant variation between studies, their findings show that since the 2002 introduction of the 'Payment by Results' program, accuracy has improved, and for primary discharge diagnoses accuracy was 96.0%.[12]

eAppendix 5: Validity of causes of death records

The present research is reliant on the accuracy of death certificates. The validity of death records underlying our study has therefore been carefully assessed. In England and Wales, information collected at death registration is normally supplied by the informant (usually a close relative of the deceased), underdoes automatic validation checks and is verified by the registrar. The cause of death is usually obtained from the 'Medical Certificate of Cause of Death', completed by a medical practitioner when the death is certified. The final underlying cause of death takes account of additional information received from medical practitioners or coroners after the death has been registered; around 40% of deaths are referred to the coroner.[13] The Office for National Statistics (ONS) collects information on all deaths that occur in England and Wales as well as deaths of all ordinary residents.[13] The dataset used in this study was restricted to patients whose record was linked to death information from ONS death certificates. Mortality data from the Office for National Statistics is used by academics, demographers and health researchers, as well as major national and international health organizations (including Public Health England, Eurostat, and the United Nations) for disease surveillance and epidemiological research. A recent study performed by the ONS has examined causes of death recorded on death certificates in five pilot areas in the UK. The study found on scrutiny of an independent medical examiner, the broad underlying cause of death (as defined by International Classification of Diseases chapter (ICD)) remained unchanged in 88 per cent of cases.[14] In the present study, the 22 ICD chapters were grouped into higher-level disease categories (respectively 9 and 11 disease categories for death and hospitalizations), so that consistency is likely to be even higher than in the aforementioned ONS study. Moreover, no national coding reform has been introduced over the study period and there is no evidence to suggest recording practices to have changed considerably over time or by age, sex, and socioeconomic sub-groups.[13] In light of this information, we conclude that UK death certificates data may present some level of inaccuracy that must be taken into account in the design and interpretation of studies relying on death registration data; yet that the information is appropriate for the study long-term temporal trends of cause-specific mortality in large populations.

© 2019 Conrad N et al. *JAMA Cardiology*.

eAppendix 6: Literature review

We searched Pubmed for reports published from 1 January 2012 to 15 February 2019 that included "heart failure" and "mortality" in their title, reviewed references from clinical practice guidelines and consulted with experts for relevant studies. We found numerous studies that investigated mortality following a hospital admission for heart failure, though few studies reported survival rates after incident diagnosis. In an attempt to compare various reports, we selected studies that reported 1-year mortality rates following a new diagnosis of heart failure and (i) referred to European or North American cohorts, (ii) included at least 1,000 patients, (iii) were not restricted to clinical trials, special care management programs, certain age-groups or associated conditions, and (iv) reported trends over time. A few relevant studies were identified (**eTable 1**). Overall these reported improvements in mortality up to around 2005 but stable rates thereafter, despite increasing uptake of new treatments, in particular beta-blockers. Most studies confined investigations to all-cause mortality, with only one study distinguishing between cardiovascular and non-cardiovascular mortality. No study investigated underlying patterns and cause-specific mortality.

© 2019 Conrad N et al. *JAMA Cardiology*.

eTable 1: Selected studies reporting heart failure mortality rates following incident heart failure.

Study	Country	Data collection period	Size	Design	Case identification	1-year mortality rates: All (trend)	1-year mortality rates: Stratified (trend)		Comments
Taylor 2019[15]	UK	2000<br>2017	55,959	Retrospective cohort	General practice consultation	25.8%<br>19.2% (↓)			The study reports modest improvements in survival (6.6% over 17 years).
Taylor 2017[16]	UK	1998-2012	54,313	Retrospective cohort	General practice consultation	18.7% (←→)			The study presents crude mortality rates.
Gerber 2015 (Olmsted County)[17]	USA	2000-2010	2,762	Prospective cohort	Outpatient and hospital discharge records	20.2% (←→)	**CVD**<br>9% (←→)	**Non-CVD**<br>11% (←→)	The study reports temporal trends with no decline in mortality rates over time.
Yeung 2012[18]	Canada	1997<br>2007	419,551	Retrospective cohort	Outpatient and hospital discharge records	27%<br>25% (↓)	**Inpatients**<br>36%<br>34% (←→)	**Outpatients**<br>18%<br>16% (↓)	Mortality rates presented are age-sex-standardized to the 1991 Ontario population aged > 20 years, and risk-adjusted to patients' comorbidity profiles.
Gomez-Soto 2011[19]	Spain	2000<br>2007	4,793	Prospective cohort	Diagnosis by GP or hospital admission	31%*<br>29%* (↓)	**Men**<br>35%<br>34% (↓)	**Women**<br>27%<br>24% (↓)	Risk-adjusted mortality rates accounting for age at incidence, comorbidities, type of heart failure, and source of incident diagnosis (inpatient vs. outpatient).
Roger 2004 (Olmsted County)[20]	USA	1979-1984<br>1985-1990<br>1991-1995<br>1996-2000	4,537	Prospective cohort	Outpatient and hospital discharge records	25%*<br>23%*<br>23%*<br>19%* (↓)	**Men**<br>30%<br>26%<br>25%<br>21% (↓)	**Women**<br>20%<br>19%<br>20%<br>17% (↓)	Mortality estimates after onset of heart failure among men and women aged 75 years. Trends compare the last to the first reporting period.
Levy 2002 (Framingham)[21]	USA	1950–1969<br>1970–1979<br>1980–1989<br>1990–1999	1,075	Prospective cohort	Population based screening	29%*<br>35%*<br>30%*<br>26%* (↓)	**Men**<br>30%<br>41%<br>33%<br>28% (↓)	**Women**<br>28%<br>28%<br>27%<br>24% (↓)	Mortality rates presented refer to men and women aged 65 to 74 years, and are adjusted for age. Trends are adjusted for age, type of heart failure and comorbidities. Trends compare the last to the first reporting period.

Abbreviations: Heart Failure (HF), General Practice (GP), United Kingdom (UK), United States of America (USA), Cardiovascular (CVD). Trends: ←→ indicates stable trend, ↓ indicates declining trend, and O indicates trends are not reported. * Overall rates are not reported; To allow overall comparison, we present estimates as the average of men and women rates. For study selection criteria, please refer to **text S3**.

© 2019 Conrad N et al. *JAMA Cardiology*.

eTable 2: Diagnostic codes that refer to a new diagnosis of heart failure

A. ICD-10 codes used in hospital records

Code	Description
I50.0	Congestive heart failure
I50.1	Left ventricular failure
I50.9	Heart failure, unspecified
I42.0	Dilated cardiomyopathy (Congestive cardiomyopathy)
I42.9	Cardiomyopathy, unspecified
I11.0	Hypertensive heart disease with (congestive) heart failure
I25.5	Ischaemic cardiomyopathy
I13.2	Hypertensive heart and renal disease with both (congestive) heart failure and renal failure
I13.0	Hypertensive heart and renal disease with (congestive) heart failure

B. Read codes used in general practice records

Medcode	Read Code	Description
884	G581.00	Left ventricular failure
2062	G58..00	Heart failure
2906	G580.11	Congestive cardiac failure
398	G580.00	Congestive heart failure
8966	G5yy900	Left Ventricular Systolic Dysfunction
3204	G55..00	Cardiomyopathy
11284	585f.00	Echocardiogram shows left ventricular systolic dysfunction
1223	G58..11	Cardiac failure
5942	G581.13	Impaired left ventricular function
7251	33BA.00	Impaired Left Ventricular Function
9913	1O1..00	Heart failure confirmed
12550	G5yyA00	Left Ventricular Diastolic dysfunction
8010	G551.00	Hypertrophic obstructive cardiomyopathy
5695	G41z.11	Chronic cor pulmonale
4024	G58z.00	Heart failure NOS
11351	585g.00	Echo shows LVDD
7535	G554400	Primary dilated cardiomyopathy
13189	662g.00	New York Heart Association classification - class II
5255	G581000	Acute left ventricular failure
10079	G580.12	Right heart failure
3499	G554300	Hypertrophic non-obstructive cardiomyopathy
16383	1O1..00	Heart failure confirmed
9524	G580.14	Biventricular failure
7320	G343.00	Ischaemic cardiomyopathy
17278	G58z.12	Cardiac failure NOS
19066	662h.00	New York Heart Association classification - class III
27964	G582.00	Acute heart failure
22993	G55z.00	Cardiomyopathy NOS
107397	G5yyD00	Left ventricular cardiac dysfunction
18853	662f.00	NYHA class f - i
101138	G583.00	Heart failure with normal ejection fraction

© 2019 Conrad N et al. *JAMA Cardiology*.

Medcode	Read Code	Description
32671	G580100	Chronic congestive heart failure
4915	G555.00	Alcoholic cardiomyopathy
10154	G580.13	Right ventricular failure
9402	G55y.11	Secondary dilated cardiomyopathy
27884	G580200	Decompensated cardiac failure
32898	8H2S.00	Admit heart failure emergency
21852	G554200	Familial cardiomyopathy
106897	G583.12	Heart failure with preserved ejection fraction
104275	G584.00	Right ventricular failure
5141	G554000	Congestive cardiomyopathy
11424	G580300	Compensated cardiac failure
97780	G559.00	Arrhythmogenic right ventricular cardiomyopathy
101137	G583.11	HFNEF - heart failure with normal ejection fraction
106008	8CMW800	Heart failure clinical pathway
94870	G580400	Congestive heart failure due to valvular disease
27683	G558100	Cardiomyopathy in myotonic dystrophy
70648	Gyu5M00	Other hypertrophic cardiomyopathy
22262	G1yz100	Rheumatic left ventricular failure
51214	662i.00	New York Heart Association classification - class IV
106198	661M500	Heart failure self-management plan agreed
103732	8CMK.00	Has heart failure management plan
62718	G21z100	Hypertensive heart disease NOS with CCF
52127	G211100	Benign hypertensive heart disease with CCF
21837	G232.00	Hypertensive heart & renal dis with (congestive) heart failure
105542	8CeC.00	Preferred place of care for next exacerbation HF

***Abbreviations**: 'HF-REF' = heart failure with reduced ejection fraction (highlighted in blue); 'HF-UNS' = heart failure with unspecified ejection fraction. 'NHFA' identifies those codes used by the National Heart Failure Audit to identify patients with a heart failure diagnosis from hospital discharge records. 'QOF HF' identifies those codes used by the 2014 Quality and Outcomes Framework to identify patients with a heart failure diagnosis from general practice records. 'QOF LVSD' refers to codes used by the 2014 Quality and Outcomes Framework to identify patients with a left ventricular systolic dysfunction diagnosis from general practice records.*

© 2019 Conrad N et al. *JAMA Cardiology*.

eTable 3: Diagnostic codes that refer to pre-existing heart failure

Code Type	Medcode	Readcode	Description
READ	95021	9N4s.00	Did not attend practice nurse heart failure clinic
READ	24503	8B29.00	Cardiac failure therapy
READ	95835	679X.00	Heart failure education
READ	26115	8HHb.00	Referral to heart failure nurse
READ	5155	23E1.00	O/E - pulmonary oedema
READ	90935	9hH..00	Exception reporting: heart failure quality indicators
READ	30749	9hH0.00	Excepted heart failure quality indicators: Patient unsuitable
READ	34213	9h1..00	Exception reporting: LVD quality indicators
READ	11613	9h11.00	Excepted from LVD quality indicators: Patient unsuitable
READ	28649	9h12.00	Excepted from LVD quality indicators: Informed dissent
READ	15058	14A6.00	H/O: heart failure
READ	46912	14AM.00	H/O: Heart failure in last year
READ	83502	662p.00	Heart failure 6-month review
READ	12366	662T.00	Congestive heart failure monitoring
READ	30779	662W.00	Heart failure annual review
READ	32945	8CL3.00	Heart failure care plan discussed with patient
READ	17851	8HBE.00	Heart failure follow-up
READ	70619	8HHz.00	Referral to heart failure exercise programme
READ	71235	8Hk0.00	Referred to heart failure education group
READ	64062	9hH1.00	Excepted heart failure quality indicators: Informed dissent
READ	32911	9Or..00	Heart failure monitoring administration
READ	19380	9Or0.00	Heart failure review completed
READ	90193	9Or1.00	Heart failure monitoring telephone invite
READ	90192	9Or2.00	Heart failure monitoring verbal invite
READ	72965	9Or3.00	Heart failure monitoring first letter
READ	72386	9Or4.00	Heart failure monitoring second letter
READ	89650	9Or5.00	Heart failure monitoring third letter
READ	18793	9On..00	Left ventricular dysfunction monitoring administration
READ	60710	9On0.00	Left ventricular dysfunction monitoring first letter
READ	60721	9On1.00	Left ventricular dysfunction monitoring second letter
READ	72341	9On2.00	Left ventricular dysfunction monitoring third letter
READ	92305	9On3.00	Left ventricular dysfunction monitoring verbal invite
READ	96484	9On4.00	Left ventricular dysfunction monitoring telephone invite
READ	100784	2126400	Heart Failure Resolved
READ	102585	8HgD.00	Discharge from heart failure nurse service
READ	106680	8HTL000	Referral to rapid access heart failure clinic
READ	106836	8IB8.00	Referral to heart failure exercise programme not indicated
READ	106894	8IE1.00	Referral to heart failure exercise programme declined
READ	107981	8IE0.00	Referral to heart failure education group declined
READ	42999	12CR.00	FH: Hypertrophic obstructive cardiomyopathy

© 2019 Conrad N et al. *JAMA Cardiology*.

eTable 4: Definition of disease categories for causes of deaths and hospitalizations

Causes of death	Causes of hospitalization
**Cardiovascular disorders**: ICD chapter 'Diseases of the circulatory system' (code range: I00–I99), excluding codes relating to infections.	**Heart failure:** ICD codes: I50 'Heart failure' (incl. I50.0, I50.1, I50.9), I42.0 'Dilated cardiomyopathy', I42.9 'Cardiomyopathy, unspecified', I11.0 'Hypertensive heart disease with (congestive) heart failure', I25.5 'Ischemic cardiomyopathy', I13.0 'Hypertensive heart and renal disease with (congestive) heart failure', I13.2 'Hypertensive heart and renal disease with both (congestive) heart failure and renal failure'.
	**Other cardiovascular disorders**: ICD chapter 'Diseases of the circulatory system' (code range: I00–I99), excluding codes relating to heart failure or infections.
**Neoplasms**: ICD chapter 'Neoplasms' (C00–D48).	
**Infections**: infectious and parasitic diseases, respiratory infections, urinary tract infections, and cellulitis, as defined by individual codes listed in e**Table 6**.	
**Chronic respiratory diseases**: individual codes listed in **eTable 7**.	
**Digestive diseases**: ICD chapter: 'Diseases of the digestive system' (K00–K93), excepting selected codes categorized as infections.	
**Mental and neurological disorders:** ICD chapter 'Mental and behavioral disorders' (F00–F99) and ICD chapter 'Diseases of the nervous system' (G00–G99)	
---	**Musculoskeletal disorders**: ICD chapter 'Diseases of the musculoskeletal system and connective tissue' (M00-M99).
**Injuries**: ICD chapters 'Injury, poisoning and certain other consequences of external causes' (S00–T98) and 'External causes of morbidity and mortality' (V01–Y98)	
**Kidney diseases** ICD sub-chapters 'Renal failure' (N17-N19), 'Glomerular diseases' (N00-N08), 'Renal tubulo-interstitial diseases' (N10-N16), 'Other disorders of kidney and ureter' (N25-N29).	
**Other**: any code not falling into any of the above categories.	**Other**: ICD chapter 'Symptoms, signs and abnormal clinical and laboratory findings, not elsewhere classified' (R00-R99) as well as any code not falling into any of the above categories.
In sub-group analyses, categories were grouped into cardiovascular (heart failure and other cardiovascular) causes and non-cardiovascular causes (all other categories).	

© 2019 Conrad N et al. *JAMA Cardiology*.

eTable 5: Number of deaths due to cardiovascular diseases, neoplasms, infections, and chronic respiratory diseases, by disease sub-group

	All years (n = 27,398)	2002-2004 (n=6,884)	2011-2013 (n=6,616)
**Cardiovascular diseases**			
Chronic ischemic heart disease	4,881 (34%)	1,501 (38%)	848 (29%)
Acute myocardial infarction	3,010 (21%)	868 (22%)	583 (20%)
Heart failure	1,794 (13%)	509 (13%)	295 (10%)
Cerebrovascular diseases	1,619 (11%)	396 (10%)	386 (13%)
Other forms of heart disease	3,027 (21%)	687 (17%)	862 (29%)
**Neoplasms**			
Malignant neoplasms of digestive organs	819 (22%)	219 (25%)	211 (21%)
Malignant neoplasms of respiratory and intrathoracic organs	708 (19%)	184 (21%)	190 (19%)
Malignant neoplasms of lymphoid, hematopoietic and related tissue	533 (14%)	102 (12%)	155 (16%)
Malignant neoplasms of male genital organs	374 (10%)	90 (10%)	107 (11%)
Malignant neoplasms of urinary tract	237 (6%)	53 (6%)	66 (7%)
Malignant neoplasm of breast	227 (6%)	50 (6%)	62 (6%)
Other	830 (22%)	183 (21%)	204 (21%)
**Infections**			
Influenza and pneumonia	1,915 (64%)	411 (72%)	566 (69%)
Other forms of respiratory infections	137 (5%)	13 (2%)	28 (3%)
Urinary tract infections	351 (12%)	50 (9%)	74 (9%)
Sepsis	192 (6%)	23 (4%)	59 (7%)
Intestinal infectious diseases	146 (5%)	20 (4%)	30 (4%)
Cellulitis	88 (3%)	11 (2%)	34 (4%)
Infections affecting the heart	61 (2%)	15 (3%)	14 (2%)
Tuberculosis	17 (1%)	6 (1%)	3 (0%)
Other infectious or parasitic diseases	62 (2%)	20 (4%)	18 (2%)
**Chronic respiratory diseases**			
Chronic obstructive pulmonary disease	1,907 (67%)	477 (73%)	460 (64%)
Interstitial lung disease and pulmonary sarcoidosis	493 (17%)	74 (11%)	170 (24%)
Asthma	62 (2%)	13 (2%)	15 (2%)
Pneumoconiosis	24 (1%)	4 (1%)	12 (2%)
Other chronic respiratory diseases	341 (12%)	84 (13%)	58 (8%)
**Mental health and neurological disorders**			
Dementia	386 (55%)	46 (39%)	199 (70%)
Alzheimer	85 (12%)	18 (15%)	23 (8%)
Other diseases of the nervous system	207 (30%)	47 (40%)	58 (20%)
Other mental and behavioral disorders	22 (3%)	6 (5%)	4 (1%)

© 2019 Conrad N et al. *JAMA Cardiology*.

**Injuries**			
Accidental exposure to unspecified factor	202 (43%)	37 (49%)	48 (38%)
Falls	152 (33%)	18 (24%)	44 (35%)
Complications of medical and surgical care	39 (8%)	14 (19%)	17 (14%)
Other external causes of morbidity and mortality	74 (16%)	6 (8%)	16 (13%)

N refers to the number of deaths at 1 year attributed to individual causes. Percentages refer to the total number of deaths within each disease category. Clinical codes used to identify each disease group are available upon request.

© 2019 Conrad N et al. *JAMA Cardiology*.

eTable 6: Clinical codes used to categorize causes of death or hospitalization as infections

A. Infectious diseases

ICD Code	Description
A00–B99	Certain infectious and parasitic diseases

B. Other respiratory infections, not categorized as infectious and parasitic diseases

ICD Code	Description
H65	Nonsuppurative otitis media
H65.0	Acute serous otitis media
H65.1	Other acute nonsuppurative otitis media
H65.2	Chronic serous otitis media
H65.3	Chronic mucoid otitis media
H65.4	Other chronic nonsuppurative otitis media
H66	Suppurative and unspecified otitis media
H66.0	Acute suppurative otitis media
H66.1	Chronic tubotympanic suppurative otitis media
H66.2	Chronic atticoantral suppurative otitis media
H72	Perforation of tympanic membrane
H72.0	Central perforation of tympanic membrane
H72.1	Attic perforation of tympanic membrane
H72.2	Other marginal perforations of tympanic membrane
H72.8	Other perforations of tympanic membrane
H72.9	Unspecified perforation of tympanic membrane
H73	Other disorders of tympanic membrane
H73.0	Acute myringitis
H73.1	Chronic myringitis
H73.2	Unspecified myringitis
H73.8	Other specified disorders of tympanic membrane
H73.9	Unspecified disorder of tympanic membrane
H80	Otosclerosis
H80.0	Otosclerosis involving oval window, nonobliterative
H80.1	Otosclerosis involving oval window, obliterative
H80.2	Cochlear otosclerosis
H80.8	Other otosclerosis
H80.9	Unspecified otosclerosis
H83	Other diseases of inner ear
H83.0	Labyrinthitis
H83.1	Labyrinthine fistula
H83.2	Labyrinthine dysfunction
H83.3	Noise effects on inner ear
H83.8	Other specified diseases of inner ear
H83.9	Unspecified disease of inner ear
J01	Acute sinusitis
J01.0	Acute maxillary sinusitis
J01.1	Acute frontal sinusitis
J01.2	Acute ethmoidal sinusitis
J01.3	Acute sphenoidal sinusitis
J01.4	Acute pansinusitis
J01.8	Other acute sinusitis
J01.9	Acute sinusitis, unspecified
J02.0	Streptococcal pharyngitis
J02.8	Acute pharyngitis due to other specified organisms
J02.9	Acute pharyngitis, unspecified
J03.0	Streptococcal tonsillitis
J03.8	Acute tonsillitis due to other specified organisms
J03.9	Acute tonsillitis, unspecified
J04	Acute laryngitis and tracheitis
J04.0	Acute laryngitis
J04.1	Acute tracheitis
J04.2	Acute laryngotracheitis
J04.3	Supraglottitis, unspecified
J05	Acute obstructive laryngitis [croup] and epiglottitis

© 2019 Conrad N et al. *JAMA Cardiology*.

ICD Code	Description
J05.0	Acute obstructive laryngitis [croup]
J05.1	Acute epiglottitis
J06.0	Acute laryngopharyngitis
J06.8	Other acute upper respiratory infections of multiple sites
J06.9	Acute upper respiratory infection, unspecified
J09	Influenza due to identified zoonotic or pandemic influenza virus
J10	Influenza due to other identified influenza virus
J10.0	Influenza due to oth identified influenza virus w pneumonia
J10.1	Flu due to oth ident influenza virus w oth resp manifest
J10.2	Influenza due to oth ident influenza virus w GI manifest
J10.8	Influenza due to oth ident influenza virus w oth manifest
J11	Influenza due to unidentified influenza virus
J11.0	Influenza due to unidentified influenza virus with pneumonia
J11.1	Flu due to unidentified influenza virus w oth resp manifest
J11.2	Influenza due to unidentified influenza virus w GI manifest
J11.8	Influenza due to unidentified influenza virus w oth manifest
J12	Viral pneumonia, not elsewhere classified
J12.0	Adenoviral pneumonia
J12.1	Respiratory syncytial virus pneumonia
J12.2	Parainfluenza virus pneumonia
J12.3	Human metapneumovirus pneumonia
J12.8	Other viral pneumonia
J12.9	Viral pneumonia, unspecified
J13	Pneumonia due to Streptococcus pneumoniae
J14	Pneumonia due to Haemophilus influenzae
J15	Bacterial pneumonia, not elsewhere classified
J15.0	Pneumonia due to Klebsiella pneumoniae
J15.1	Pneumonia due to Pseudomonas
J15.2	Pneumonia due to staphylococcus
J15.3	Pneumonia due to streptococcus, group B
J15.4	Pneumonia due to other streptococci
J15.5	Pneumonia due to Escherichia coli
J15.6	Pneumonia due to other aerobic Gram-negative bacteria
J15.7	Pneumonia due to Mycoplasma pneumoniae
J15.8	Pneumonia due to other specified bacteria
J15.9	Unspecified bacterial pneumonia
J16.0	Chlamydial pneumonia
J16.8	Pneumonia due to other specified infectious organisms
J17	Pneumonia in diseases classified elsewhere
J17.0	Pneumonia in bacterial diseases classified elsewhere
J17.1	Pneumonia in viral diseases classified elsewhere
J17.2	Pneumonia in mycoses
J17.3	Pneumonia in parasitic diseases
J17.8	Pneumonia in other diseases classified elsewhere
J18	Pneumonia, unspecified organism
J18.0	Bronchopneumonia, unspecified organism
J18.1	Lobar pneumonia, unspecified organism
J18.2	Hypostatic pneumonia, unspecified organism
J18.8	Other pneumonia, unspecified organism
J18.9	Pneumonia, unspecified organism
J20	Acute bronchitis
J20.0	Acute bronchitis due to Mycoplasma pneumoniae
J20.1	Acute bronchitis due to Hemophilus influenzae
J20.2	Acute bronchitis due to streptococcus
J20.3	Acute bronchitis due to coxsackievirus
J20.4	Acute bronchitis due to parainfluenza virus
J20.5	Acute bronchitis due to respiratory syncytial virus
J20.6	Acute bronchitis due to rhinovirus
J20.7	Acute bronchitis due to echovirus
J20.8	Acute bronchitis due to other specified organisms
J20.9	Acute bronchitis, unspecified
J21	Acute bronchiolitis
J21.0	Acute bronchiolitis due to respiratory syncytial virus

© 2019 Conrad N et al. *JAMA Cardiology*.

ICD Code	Description
J21.1	Acute bronchiolitis due to human metapneumovirus
J21.8	Acute bronchiolitis due to other specified organisms
J21.9	Acute bronchiolitis, unspecified
J22	Unspecified acute lower respiratory infection
J32.9	Chronic sinusitis, unspecified
J40	Bronchitis, not specified as acute or chronic
J85.1	Abscess of lung with pneumonia
J86.9	Pyothorax without fistula
K67.3	Tuberculous peritonitis
K93.0	Tuberculous disorders of intestines, peritoneum and mesenteric glands
N74.1	Female tuberculous pelvic inflammatory disease

C. Urinary tract infections

ICD Code	Description
N11	Chronic tubulointerstitial nephritis
N11.0	Nonobstructive refluxassociated chronic pyelonephritis
N11.1	Chronic obstructive pyelonephritis
N11.8	Other chronic tubulointerstitial nephritis
N11.9	Chronic tubulointerstitial nephritis, unspecified
N15	Other renal tubulointerstitial diseases
N15.0	Balkan nephropathy
N15.1	Renal and perinephric abscess
N30	Cystitis
N30.0	Acute cystitis
N30.1	Interstitial cystitis (chronic)
N30.2	Other chronic cystitis
N30.3	Trigonitis
N30.4	Irradiation cystitis
N30.8	Other cystitis
N30.9	Cystitis, unspecified
N34	Urethritis and urethral syndrome
N34.0	Urethral abscess
N34.1	Nonspecific urethritis
N34.2	Other urethritis
N34.3	Urethral syndrome, unspecified
N39.0	Urinary tract infection, site not specified

D. Other infectious diseases

ICD Code	Description
G00	Bacterial meningitis, not elsewhere classified
G00.0	Hemophilus meningitis
G00.1	Pneumococcal meningitis
G00.2	Streptococcal meningitis
G00.3	Staphylococcal meningitis
G00.8	Other bacterial meningitis
G00.9	Bacterial meningitis, unspecified
G03	Meningitis due to other and unspecified causes
G03.0	Nonpyogenic meningitis
G03.1	Chronic meningitis
G03.2	Benign recurrent meningitis [Mollaret]
G03.8	Meningitis due to other specified causes
G03.9	Meningitis, unspecified
G04	Encephalitis, myelitis and encephalomyelitis
G04.0	Acute disseminated encephalitis and encephalomyelitis (ADEM)
G04.1	Tropical spastic paraplegia
G04.2	Bacterial meningoencephalitis and meningomyelitis, NEC
G04.3	Acute necrotizing hemorrhagic encephalopathy
G04.8	Other encephalitis, myelitis and encephalomyelitis
G04.9	Encephalitis, myelitis and encephalomyelitis, unspecified
H70.1	Chronic mastoiditis

© 2019 Conrad N et al. *JAMA Cardiology*.

ICD Code	Description
I00	Rheumatic fever without mention of heart involvement
I01	Rheumatic fever with heart involvement
I01.0	Acute rheumatic pericarditis
I01.1	Acute rheumatic endocarditis
I01.2	Acute rheumatic myocarditis
I01.8	Other acute rheumatic heart disease
I01.9	Acute rheumatic heart disease, unspecified
I02	Rheumatic chorea
I02.0	Rheumatic chorea with heart involvement
I02.9	Rheumatic chorea without heart involvement
I30	Acute pericarditis
I30.0	Acute nonspecific idiopathic pericarditis
I30.1	Infective pericarditis
I30.8	Other forms of acute pericarditis
I30.9	Acute pericarditis, unspecified
I33	Acute and subacute endocarditis
I33.0	Acute and subacute infective endocarditis
I33.9	Acute endocarditis, unspecified
I40	Acute myocarditis
I40.0	Infective myocarditis
I40.1	Isolated myocarditis
I40.8	Other acute myocarditis
I40.9	Acute myocarditis, unspecified
L03	Cellulitis

© 2019 Conrad N et al. *JAMA Cardiology*.

eTable 7: Clinical codes used to categorize causes of death or hospitalization as chronic respiratory diseases

ICD Code	Description
D86	Sarcoidosis
D86.0	Sarcoidosis of lung
D86.1	Sarcoidosis of lymph nodes
D86.2	Sarcoidosis of lung with sarcoidosis of lymph nodes
D86.8	Sarcoidosis of other sites
D86.9	Sarcoidosis, unspecified
J38.0	Paralysis of vocal cords and larynx
J38.6	Stenosis of larynx
J39.0	Retropharyngeal and parapharyngeal abscess
J39.2	Other diseases of pharynx
J39.8	Other specified diseases of upper respiratory tract
J41	Simple and mucopurulent chronic bronchitis
J41.0	Simple chronic bronchitis
J41.1	Mucopurulent chronic bronchitis
J41.8	Mixed simple and mucopurulent chronic bronchitis
J42	Unspecified chronic bronchitis
J43	Emphysema
J43.0	Unilateral pulmonary emphysema [MacLeod s syndrome]
J43.1	Panlobular emphysema
J43.2	Centrilobular emphysema
J43.8	Other emphysema
J43.9	Emphysema, unspecified
J44	Other chronic obstructive pulmonary disease
J44.0	Chronic obstructive pulmonary disease with acute lower respiratory infection
J44.1	Chronic obstructive pulmonary disease with (acute) exacerbation
J44.8	Other specified chronic obstructive pulmonary disease
J44.9	Chronic obstructive pulmonary disease, unspecified
J45	Asthma
J45.0	Predominantly allergic asthma
J45.1	Nonallergic asthma
J45.2	Mild intermittent asthma
J45.3	Mild persistent asthma
J45.4	Moderate persistent asthma
J45.5	Severe persistent asthma
J45.8	Mixed asthma
J45.9	Other and unspecified asthma
J46	Status asthmaticus
J47	Bronchiectasis
J47.0	Bronchiectasis with acute lower respiratory infection
J47.1	Bronchiectasis with (acute) exacerbation
J47.9	Bronchiectasis, uncomplicated
J60	Coalworker s pneumoconiosis
J61	Pneumoconiosis due to asbestos and other mineral fibers
J62.0	Pneumoconiosis due to talc dust
J62.8	Pneumoconiosis due to other dust containing silica
J63	Pneumoconiosis due to other inorganic dusts
J63.0	Aluminosis (of lung)
J63.1	Bauxite fibrosis (of lung)
J63.2	Berylliosis
J63.3	Graphite fibrosis (of lung)
J63.4	Siderosis
J63.5	Stannosis
J63.8	Pneumoconiosis due to other specified inorganic dusts
J64	Unspecified pneumoconiosis
J67.0	Farmer lung
J67.9	Hypersensitivity pneumonitis due to unspecified organic dust
J69.0	Pneumonitis due to food and vomit
J81	Pulmonary oedema
J82	Pulmonary eosinophilia, not elsewhere classified
J84	Other interstitial pulmonary diseases

© 2019 Conrad N et al. *JAMA Cardiology*.

ICD Code	Description
J84.0	Alveolar and parietoalveolar conditions
J84.1	Other interstitial pulmonary diseases with fibrosis
J84.2	Lymphoid interstitial pneumonia
J84.8	Other specified interstitial pulmonary diseases
J84.9	Interstitial pulmonary disease, unspecified
J90	Pleural effusion, not elsewhere classified
J92.0	Pleural plaque with presence of asbestos
J93.1	Other spontaneous pneumothorax
J93.9	Pneumothorax, unspecified
J94.1	Fibrothorax
J94.8	Other specified pleural conditions
J96.1	Chronic respiratory failure
J96.9	Respiratory failure, unspecified
J98.1	Pulmonary collapse
J98.4	Other disorders of lung
J98.8	Other specified respiratory disorders
J98.9	Respiratory disorder, unspecified

© 2019 Conrad N et al. *JAMA Cardiology*.

eFigure 1: All-cause and cause-specific mortality rates at 1-year following incident heart failure, by age and sex.

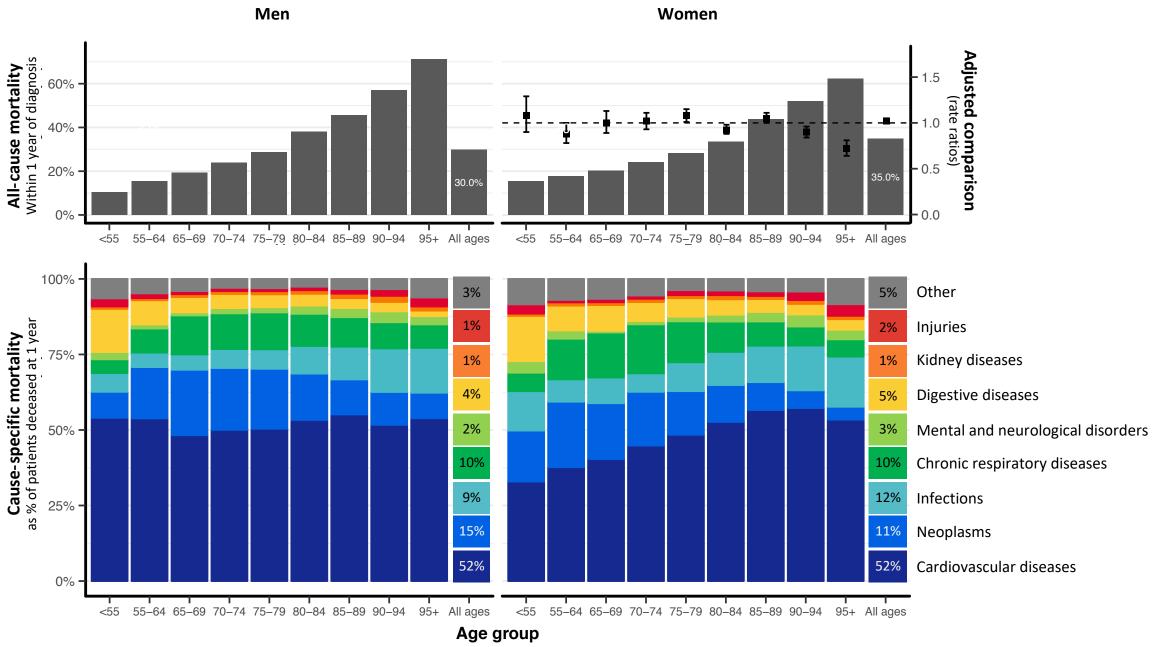

Crude rates of all-cause mortality rates at 1 year following incident heart failure diagnosis, as well as cause-specific mortality presented as a proportion of patients deceased at 1-year. Adjusted comparison present rate ratios for all-cause mortality at 1 year in women versus men from multivariable Poisson regression models adjusting for year of diagnosis, socioeconomic status, region, and 17 baseline comorbidities.

© 2019 Conrad N et al. *JAMA Cardiology*.

eFigure 2: Differences in hospital admissions within a year of incident heart failure, by sex, socioeconomic status and diagnosis care setting.

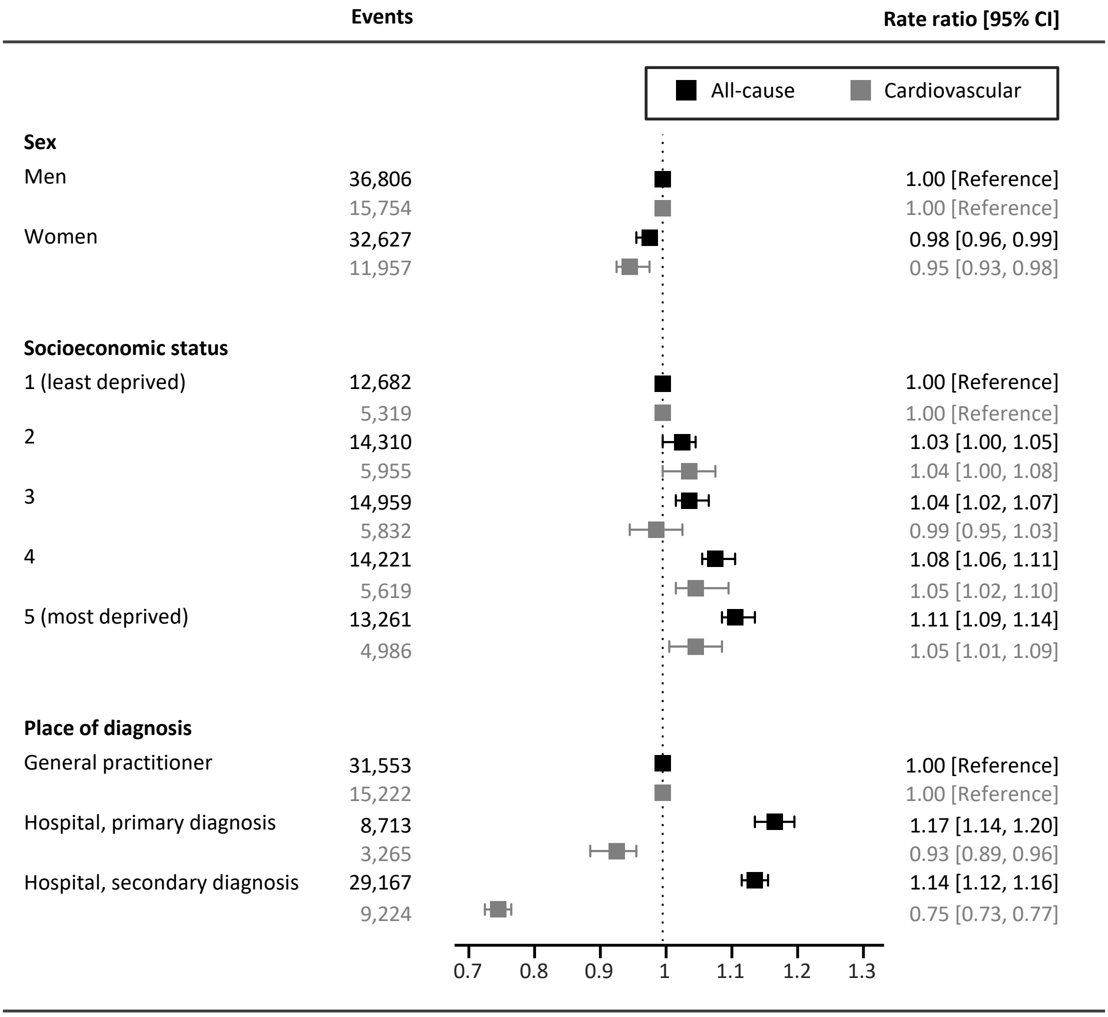

Hospital admissions refer to the number of admissions per patient-years at risk within a year of incident heart failure diagnosis. Rate ratios and 95% confidence intervals (CI) from multivariable Poisson regression models accounting for year of diagnosis, age (as a continuous variable), sex, socioeconomic status, region, and 17 baseline comorbidities. Socioeconomic status refers to Index of Multiple Deprivation 2015 quintile, with 1 referring to the most affluent and 5 to the most deprived socioeconomic quintile.

© 2019 Conrad N et al. *JAMA Cardiology*.

eFigure 3: Temporal trends in one-year mortality rates following incident heart failure, by age group.

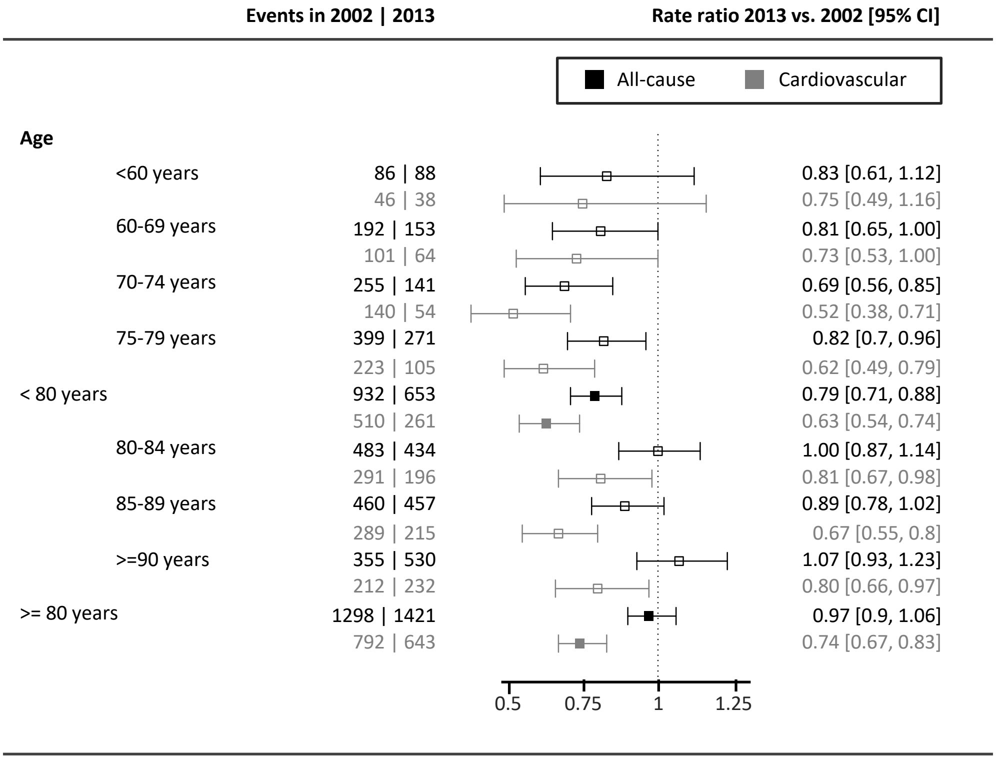

Rate ratios and 95% confidence intervals (CI) comparing 2013 and 2002, from multivariable Poisson regression models accounting for year of diagnosis, age (as a continuous variable), sex, socioeconomic status, region, and 17 baseline comorbidities.

© 2019 Conrad N et al. *JAMA Cardiology*.

References

1. Office for National Statistics. *Guidance for Doctors Completing Medical Certificates of Cause of Death in England and Wales.*; 2018. https://assets.publishing.service.gov.uk/government/uploads/system/uploads/attachment_data/file/757010/guidance-for-doctors-completing-medical-certificates-of-cause-of-death.pdf. Accessed August 1, 2019.

2. Berlin A. Death certification: topical tips for GPs. *London J Prim Care (Abingdon)*. 2009;2(2):130-137. doi:10.1080/17571472.2009.11493267

3. CALIBER Research Portal. https://www.caliberresearch.org/ portal/. Accessed February 1, 2017.

4. Department for Communities and Local Government (DCLG). *The English Index of Multiple Deprivation 2015: Guidance.*; 2015. https://www.gov.uk/government/statistics/english-indices-of-deprivation-2015. Accessed February 8, 2017.

5. Herrett E, Thomas SL, Schoonen WM, Smeeth L, Hall AJ. Validation and validity of diagnoses in the General Practice Research Database: a systematic review. *Br J Clin Pharmacol*. 2010;69(1):4-14. doi:10.1111/j.1365-2125.2009.03537.x

6. Johansson S, Wallander M-A, Ruigómez A, Rodríguez LAG. Incidence of newly diagnosed heart failure in UK general practice. *Eur J Heart Fail*. 2001;3(2):225-231. doi:10.1016/S1388-9842(00)00131-8

7. Quint JK, Müllerova H, DiSantostefano RL, et al. Validation of chronic obstructive pulmonary disease recording in the Clinical Practice Research Datalink (CPRD-GOLD). *BMJ Open*. 2014;4(7):e005540. doi:10.1136/bmjopen-2014-005540

8. Primary Care Domain ND. *Quality and Outcomes Framework Report, England 2015-16.*; 2016. http://content.digital.nhs.uk/qof. Accessed February 27, 2017.

9. Mitchell P, Marle D, Donkor A, et al. *National Heart Failure Audit 2013-2014.*; 2015. https://www.ucl.ac.uk/nicor/audits/heartfailure/documents/annualreports/hfannual13-14-updated.pdf. Accessed October 12, 2016.

10. National Institute for Clinical Excellence (NICE). *Management of Chronic Heart Failure in Adults in Primary and Secondary Care. Clinical Guideline 5.*; 2003.

11. National Institute for Health and Clinical Excellence (NICE). *Chronic Heart Failure: Management of Chronic Heart Failure in Adults in Primary and Secondary Care (CG108).*; 2010. http://guidance.nice.org.uk/CG108/Guidance.

12. Burns EM, Rigby E, Mamidanna R, et al. Systematic review of discharge coding accuracy. *J Public Health (Oxf)*. 2012;34(1):138-148. doi:10.1093/pubmed/fdr054

13. Office for National Statistics. *Mortality Statistics in England and Wales - Quality and Methodology Information.*; 2018. https://www.ons.gov.uk/peoplepopulationandcommunity/birthsdeathsandmarriages/deaths/methodologies/mortalitystatisticsinenglandandwalesqmi. Accessed May 10, 2019.

14. Office for National Statistics. *Death Certification Reform: A Case Study on the Potential Impact on Mortality Statistics, England and Wales.*; 2012. https://assets.publishing.service.gov.uk/government/uploads/system/uploads/attachment_data/file/217021/dcp171778_288141.pdf. Accessed July 30, 2019.

15. Taylor CJ, Ordóñez-Mena JM, Roalfe AK, et al. Trends in survival after a diagnosis of heart failure in the United Kingdom 2000-2017: population based cohort study. *BMJ*. 2019;364:l223. doi:10.1136/bmj.l223

16. Taylor CJ, Ryan R, Nichols L, Gale N, Hobbs R, Marshall T. Survival following a diagnosis of heart failure in primary care. *Fam Pract*. 2017;(5):1-8. doi:10.1093/fampra/cmw145

17. Gerber Y, Weston SA, Redfield MM, et al. A contemporary appraisal of the heart failure epidemic in olmsted county, Minnesota, 2000 to 2010. *JAMA Intern Med*. 2015;175(6):996-1004. doi:10.1001/jamainternmed.2015.0924

18. Yeung DF, Boom NK, Guo H, Lee DS, Schultz SE, Tu J V. Trends in the incidence and outcomes of

© 2019 Conrad N et al. *JAMA Cardiology*.

heart failure in Ontario, Canada: 1997 to 2007. *CMAJ*. 2012;184(14):E765-73. doi:10.1503/cmaj.111958

19. Gomez-Soto FM, Andrey JL, Garcia-Egido AA, et al. Incidence and mortality of heart failure: A community-based study. *Int J Cardiol*. 2011;151(1):40-45. doi:10.1016/j.ijcard.2010.04.055

20. Roger VL, Weston SA, Redfield MM, et al. Trends in heart failure incidence and survival in a community-based population. *JAMA*. 2004;292(3):344-350. doi:10.1001/jama.292.3.344 292/3/344 [pii]

21. Levy D, Kenchaiah S, Larson MG, et al. Long-Term Trends in the Incidence of and Survival with Heart Failure. *N Engl J Med*. 2002;347(18):1397-1402. doi:10.1056/NEJMoa020265

© 2019 American Medical Association. All rights reserved.
